# Roles for Plant Mitochondrial Alternative Oxidase Under Normoxia, Hypoxia, and Reoxygenation Conditions

**DOI:** 10.3389/fpls.2020.00566

**Published:** 2020-05-15

**Authors:** Jayamini Jayawardhane, Devin W. Cochrane, Poorva Vyas, Natalia V. Bykova, Greg C. Vanlerberghe, Abir U. Igamberdiev

**Affiliations:** ^1^Department of Biology, Memorial University of Newfoundland, St. John’s, NL, Canada; ^2^Morden Research and Development Centre, Agriculture and Agri-Food Canada, Morden, MB, Canada; ^3^Department of Biological Sciences, University of Toronto Scarborough, Toronto, ON, Canada; ^4^Department of Cell and Systems Biology, University of Toronto Scarborough, Toronto, ON, Canada

**Keywords:** alternative oxidase, hypoxia, mitochondria, nitric oxide, reactive oxygen species, reoxygenation

## Abstract

Alternative oxidase (AOX) is a non-energy conserving terminal oxidase in the plant mitochondrial electron transport chain (ETC) that has a lower affinity for oxygen than does cytochrome (cyt) oxidase. To investigate the role(s) of AOX under different oxygen conditions, wild-type (WT) *Nicotiana tabacum* plants were compared with AOX knockdown and overexpression plants under normoxia, hypoxia (near-anoxia), and during a reoxygenation period following hypoxia. Paradoxically, under all the conditions tested, the AOX amount across plant lines correlated positively with leaf energy status (ATP/ADP ratio). Under normoxia, AOX was important to maintain respiratory carbon flow, to prevent the mitochondrial generation of superoxide and nitric oxide (NO), to control lipid peroxidation and protein *S*-nitrosylation, and possibly to reduce the inhibition of cyt oxidase by NO. Under hypoxia, AOX was again important in preventing superoxide generation and lipid peroxidation, but now contributed positively to NO amount. This may indicate an ability of AOX to generate NO under hypoxia, similar to the nitrite reductase activity of cyt oxidase under hypoxia. Alternatively, it may indicate that AOX activity simply reduces the amount of superoxide scavenging of NO, by reducing the availability of superoxide. The amount of inactivation of mitochondrial aconitase during hypoxia was also dependent upon AOX amount, perhaps through its effects on NO amount, and this influenced carbon flow under hypoxia. Finally, AOX was particularly important in preventing nitro-oxidative stress during the reoxygenation period, thereby contributing positively to the recovery of energy status following hypoxia. Overall, the results suggest that AOX plays a beneficial role in low oxygen metabolism, despite its lower affinity for oxygen than cytochrome oxidase.

## Introduction

The plant mitochondrial electron transport chain (ETC) is branched so that electrons in the ubiquinone pool can pass to oxygen via the usual cytochrome (cyt) pathway [involving Complex III, cyt *c* and cyt oxidase (EC 1.9.3.1)] or via alternative oxidase (AOX; EC 1.10.3.11) (Vanlerberghe, 2013; Del-Saz et al., 2018; Selinski et al., 2018). Electron flow from ubiquinol to oxygen via the cyt pathway is coupled to proton translocation and hence contributes to the synthesis of ATP. However, electron flow from ubiquinol to oxygen via AOX is not coupled to proton translocation, hence not contributing to ATP synthesis. Having two pathways with different energy yields provides a means to maintain carbon, redox, and/or energy balance in response to the changing demands on metabolism imposed by internal (e.g., developmental) and external (e.g., environmental stress) factors (Millar et al., 1998; Ribas-Carbo et al., 2005; Sieger et al., 2005; Giraud et al., 2008; Smith et al., 2009; Chai et al., 2010; Cvetkovska and Vanlerberghe, 2012b; Dahal and Vanlerberghe, 2017, 2018a).

One consequence of AOX respiration is that it can influence the generation of reactive oxygen species (ROS) and reactive nitrogen species by the mitochondrial ETC. For example, studies have shown that chemical inhibition or genetic knockdown/knockout of AOX can increase mitochondrial amounts of such species, including superoxide (O_2_^–^) and nitric oxide (NO) (Purvis, 1997; Maxwell et al., 1999; Parsons et al., 1999; Giraud et al., 2008; Smith et al., 2009; Cvetkovska and Vanlerberghe, 2012a; Alber et al., 2017).

Studies using different plant species and tissues have shown that AOX amount can change in response to hypoxia, anoxia, or reoxygenation after a low oxygen treatment (Amor et al., 2000; Tsuji et al., 2000; Klok et al., 2002; Szal et al., 2003; Millar et al., 2004; Liu et al., 2005; Kreuzwieser et al., 2009; Skutnik and Rychter, 2009; Narsai et al., 2011; Gupta et al., 2012; Vergara et al., 2012; Rivera-Contreras et al., 2016; Vishwakarma et al., 2018; Guan et al., 2019; Panozzo et al., 2019; Wany et al., 2019). The majority, but not all, of these studies indicate an increase of AOX transcript, protein and/or maximum activity (capacity) under such conditions. These findings hint at an increased role for AOX during conditions of low or fluctuating oxygen concentrations. On the other hand, AOX has a lower affinity for oxygen than cyt oxidase, suggesting that it may be a less prominent component of respiration at low oxygen concentration (Millar et al., 1994; Ribas-Carbo et al., 1994; Affourtit et al., 2001). The discrepancy between these two views may be due to another complicating factor, which is the potential for NO production in the mitochondrion at low oxygen (Gupta et al., 2018). For example, at low oxygen, cyt oxidase displays a nitrite reductase activity, generating NO from nitrite (Castello et al., 2006; Poyton et al., 2009). Further, this NO can then inhibit the oxidase activity of the enzyme (Poyton et al., 2009). On the other hand, AOX is NO-resistant, continuing to function effectively as an oxidase in the presence of NO (Millar and Day, 1997). Hence, at low oxygen, there could be a shift in the consumption of oxygen away from cyt oxidase and toward AOX, despite the lower affinity for oxygen of AOX compared to cyt oxidase.

In the current study, we compared wild-type (WT) tobacco plants to both AOX knockdown and overexpression plants under different oxygen conditions, including normoxia, hypoxia (near-anoxia), and reoxygenation following hypoxia. Our results show that AOX amount influences leaf carbon and energy metabolism under all these conditions. This indicates that AOX has role(s) in low oxygen metabolism, despite its lower affinity for oxygen than cyt oxidase.

## Materials and Methods

### Plant Material and Growth Conditions

Tobacco (*Nicotiana tabacum* L. cv. Petit Havana SR1) WT, AOX knockdown (RI9, RI29) and AOX overexpression (B7, B8) plants were grown for 1 month in controlled environment growth chambers (Model PGR-15, Conviron, Winnipeg, MB, Canada) with a 14 h photoperiod, temperature of 26°C/20°C (light/dark), relative humidity of 50%, and photosynthetic photon flux density of 150 μmol m^–2^ s^–1^. Plants were irrigated every other day and fertilized every 2 weeks with a 20-20-20 fertilizer containing 5.9% of nitrate nitrogen. The transgenic lines used in this study have been characterized earlier (Amirsadeghi et al., 2006; Wang et al., 2011; Wang and Vanlerberghe, 2013; Dahal and Vanlerberghe, 2018a). Amongst these, RI29 is the most effective knockdown (no detectable leaf AOX protein), while faint amounts of leaf AOX protein can still sometimes be detected in RI9. B8 and B7 are both effective AOX overexpressors, with B8 showing slightly higher amounts of AOX protein than B7. Previous studies have shown that under optimal (well-watered, ambient CO_2_) growth conditions, all these plant lines (WT, RI9, RI29, B7, and B8) display similar growth, respiratory rates, photosynthetic properties, and maximal cyt oxidase activities and protein amounts (Dahal and Vanlerberghe, 2017, 2018a,b; Dahal et al., 2017).

### Normoxia, Hypoxia, and Reoxygenation Treatments

To test the plants under oxygen deficiency, they were placed in a custom-built, sealed, 2 L, dark chamber for 4 h. A steady inflow of nitrogen gas was maintained at 120 mL min^–1^ (Alphagaz 1 grade having ∼0.001% oxygen) at one opening and unidirectional air valves facing outward in the openings to maintain ambient pressure within the chamber while preventing ambient air from entering (Cochrane et al., 2017). In a few experiments, the nitrogen gas contained either 0.1% or 3% oxygen. The chamber was opaque to obscure any light and contained two small openings on either side. The control plants (normoxia treatment) were flushed with air at the same flow rate in the same chamber for 4 h. Fully developed fourth or fifth leaves of the plants were harvested immediately after the treatment, frozen in liquid nitrogen and stored at −80°C for subsequent analyses. For the post-hypoxia or reoxygenation treatment, the plants were flushed with normal air for 15 and 120 min after the hypoxia treatment.

### Reactive Oxygen Species

Fresh leaf biomass (100 mg) was crushed using a mortar and pestle over ice in 1 mL of 8 M KOH and then centrifuged (15,000 *g*, 10 min, 4°C). The amount of superoxide anion (O_2_^–^) in the supernatant was then measured at 550 nm by reduction of cyt *c* using a method modified from Sun and Trumpower (2003), as described in Ma et al. (2016).

For H_2_O_2_ measurements, fresh leaf biomass (100 mg) was crushed using a mortar and pestle over ice, homogenized for 30 min in 6% trichloroacetic acid (TCA) and then centrifuged (15,000 *g*, 10 min, 4°C). H_2_O_2_ content in the supernatant was determined using a Pierce Quantitative Peroxide Assay kit (Thermo Fisher Scientific).

### Lipid and Protein Modifications

Lipid peroxidation was measured as malondialdehyde (MDA) content, using the thiobarbituric acid (TBA) method described earlier (Heath and Parker, 1968), with minor modifications. Fresh leaf biomass (250 mg) was homogenized in 5 mL of 0.1% TCA and centrifuged (10,000 *g*, 5 min, 4°C). A 1 mL aliquot of supernatant was combined with 4 mL of 20% TCA containing 0.5% TBA. This mixture was incubated (95°C, 30 min), quickly cooled in crushed ice, and centrifuged (10,000 *g*, 10 min, 4°C). The absorbance of the sample was measured spectrophotometrically at 532 and 600 nm. The non-specific absorbance at 600 nm was subtracted from the absorbance at 532 nm and the concentration of MDA was calculated using an extinction coefficient of 155 mM^–1^ cm^–1^.

The measurement of protein *S*-nitrosylation was performed by reducing R-SNO to R-SH in the presence of ascorbate and then assaying free thiol groups using 5,5’-dithiol-bis (2-nitrobenzoic acid) (DTNB) (Ma et al., 2016). Leaf biomass (100 mg) was homogenized in 1.8 mL of 50 mM HEPES (pH 8.0) containing 1 mM EDTA, 0.1 mM neocuproine, 0.2% (w/v) SDS and 0.5% (w/v) CHAPS. The homogenate was centrifuged (15,000 *g*, 10 min, 4°C) and proteins in the supernatant were precipitated by two volumes of ice cold acetone (-20°C) overnight. The protein precipitate was collected by centrifugation (15,000 *g*, 10 min, 4°C) and washed four times with chilled 70% acetone before resuspension in the same volume of extraction buffer. The protein solution was separated into two 0.9 mL samples. One sample (experimental) was combined with 50 μL of 100 mM ascorbate and the other sample (control) was combined with 50 μl of water. After incubating for 1 h at 25°C, 50 μL of 10 mM DTNB in 75 mM phosphate buffer (pH 7.0) was added to each sample, and each were then measured spectrophotometrically at 412 nm. Samples without protein were used as blanks, and the difference between experimental and control samples was used to calculate the quantity of R-SNO. The total concentration of protein was measured using Bradford reagent (Sigma-Aldrich, United States).

### Adenylates

The extraction of ATP and ADP was modified from Dordas et al. (2003) as described earlier (Cochrane et al., 2017). Fresh leaf biomass (100 mg) was rapidly frozen in liquid nitrogen and homogenized in 1 mL of 2.4 M HCl using a mortar and pestle. The homogenate was then transferred to a microcentrifuge tube, neutralized using 5 M KOH and centrifuged (20,000 *g*, 10 min, 4°C). ATP and ADP in the supernatant were then measured using a luciferase-based bioluminescent assay (EnzyLightTM ADP/ATP ratio Assay Kit ELDT-100 by BioAssay Systems, CA, United States) and FB 12 Single Tube Luminometer by Titertek-Berthold (Berthold Detection Systems, GmbH, Germany).

### NO Measurements Under Hypoxia

Leaves were detached, weighed and immediately placed in 20 mM Hepes buffer (pH 7.0) containing 50 mM sodium nitrate. When inhibitors were used, this buffer also contained either 2 mM potassium cyanide (KCN), 5 mM salicylhydroxamic acid (SHAM) or 300 μM sodium tungstate (Na_2_WO_4_). The leaves in buffer were then placed in an air tight chamber as described earlier with a constant inflow of nitrogen gas at 120 mL min^–1^ (0.001% O_2_). NO was measured by chemiluminescence detection (Planchet et al., 2005). In brief, a constant flow of measuring gas (purified air or nitrogen) at 120 mL min^–1^ was pulled through the chamber and subsequently through the chemiluminescence detector (CLD 770 AL ppt; Eco-Physics, Dürnten, Switzerland; detection limit 20 ppt; 20 s time resolution) by a vacuum pump connected to an ozone destroyer. The ozone generator of the chemiluminescence detector was supplied with dry oxygen (99%). The inflowing gas (air or nitrogen) was made NO free by passing it through a NO scrubber (Eco Physics Ltd, Switzerland). Calibration was routinely carried out with NO free air (0 ppt NO) and with various concentrations of NO (1–35 ppb) adjusted by mixing the calibration gas (500 ppb NO in nitrogen; Messer Griesheim, Darmstadt, Germany) with NO-free air. Flow controllers (Fisher Scientific) were used to adjust all gas flows.

### Aconitase (EC 4.2.1.3) Activity

Fresh leaf biomass (100 mg) was homogenized in 1 mL of cold extraction buffer (50 mM Tris–HCl [pH 7.4], 1 mM dithiothreitol, 5 mM MgCl_2_, 2 mM trisodium citrate and 0.4 M mannitol). The extract was centrifuged (3,000 *g*, 5 min, 4°C) and the supernatant was then centrifuged again (10,000 *g*, 20 min, 4°C). The pellet (containing mitochondria) was then resuspended in a buffer containing 50 mM Tris–HCl (pH 7.4), 2 mM trisodium citrate, 5 mM dithiothreitol and 100 mM MgCl_2_ and used to measure mitochondrial aconitase activity. The supernatant was also collected and used to measure cytosolic aconitase activity. Cross-contamination between the mitochondrial and cytosolic fractions was determined using marker enzymes (succinate dehydrogenase for mitochondria, lactate dehydrogenase for cytosol), as described earlier (Eprintsev et al., 2017). Cross-contamination did not exceed 10%. To measure aconitase activity, 0.1 mL of extract was added to 0.9 mL of assay buffer (50 mM Tris–HCl [pH 7.4] and 40 mM citrate). Enzyme activity was measured spectrophotometrically (240 nm) for 10 min using an Ultraspec 4300 (Biochrom). An extinction coefficient of 3.6 mM^–1^ cm^–1^ was used for cis-aconitate (Baumgart and Bott, 2011).

### Metabolomics

Metabolomics was performed using NMR analysis (Psychogios et al., 2011). Leaf tissue was flash frozen in liquid nitrogen and powdered using ceramic beads and a tabletop centrifuge. The resulting powder was homogenized in 2 M perchloric acid and incubated at room temperature for 1 h. Homogenates were neutralized on ice to pH 7 using 3 M KOH and the subsequent potassium perchlorate precipitate was removed by centrifugation (15 000 *g*, 10 min, 4 °C). Samples were then freeze-dried for 48 h before being homogenized in heavy water (D_2_O) using a ceramic mortar. After incubation for 24 h at room temperature, the solutions were centrifuged and an aliquot of the supernatant (aqueous extract) with a volume of ∼0.6 mL was placed in a vial for NMR analysis. Subsequently, 140 μL of a standard buffer solution (54% D_2_O: 46% 150 mM KH_2_PO_4_ pH 7.0 v/v containing 5.0 mM DSS-d6 (2,2-dimethyl-2-silcepentane-5-sulphonate), 5.84 mM 2-chloropyrimidine-5 carboxylate, and 0.1% NaN_3_ in H_2_O) was added to the sample. The plant sample (350 μL) was then transferred to a 3 mm SampleJet NMR tube for subsequent spectral analysis (Canam et al., 2013). All ^1^H-NMR spectra were collected on a 700 MHz Avance III (Bruker) spectrometer equipped with a 5 mm HCN Z-gradient pulsed-field gradient (PFG) cryoprobe. ^1^H-NMR spectra were acquired at 25°C using the first transient of the NOESY pre-saturation pulse sequence (noesy1dpr), chosen for its high degree of quantitative accuracy. All FID’s (free induction decays) were zero-filled to 250 K data points. The singlet produced by the DSS methyl groups was used as an internal standard for chemical shift referencing (set to 0 ppm) and for quantification. All ^1^H-NMR spectra were processed and analyzed using the Chenomx NMR Suite Professional software package version 8.1 (Chenomx Inc., Edmonton, AB, United States). The Chenomx NMR Suite software allows for qualitative and quantitative analysis of an NMR spectrum by manually fitting spectral signatures from an internal database to the spectrum. Typically, 90% of visible peaks were assigned to a compound and more than 90% of the spectral area could be routinely fit using the Chenomx spectral analysis software. Most of the visible peaks are annotated with a compound name.

### Total Phenolics and Flavonoids

Soluble phenolics and flavonoids were extracted from leaves by homogenizing in 80% (v/v) acetone with 0.2% (m/v) formic acid in a ratio of 1:10. The homogenate was then shaken (8 h, 4°C) and then centrifuged (20,000 *g*, 20 min, 4°C). The residue was extracted twice again under the same conditions and the supernatants were combined.

Total soluble phenolic content was determined using Folin-Ciocalteu reagent as described by Chandrasekara and Shahidi (2011) with modifications (Vyas et al., 2013). The Folin-Ciocalteu reagent (0.5 mL) was combined with 0.5 mL of leaf extract and mixed well. Saturated sodium carbonate solution (1 mL) was then added to neutralize the reaction. The final volume was adjusted to 10 mL by adding water and vortexing for 1 min. The samples were then kept in the dark (35 min, room temperature), and then centrifuged (4,000 *g*, 10 min). The absorbance was measured at 725 nm. Total soluble phenolic content of each sample was determined using a gallic acid standard curve and expressed as gallic acid equivalents (GAE) per leaf fresh weight.

Total flavonoid content was measured by aluminum chloride colorimetric assay (Zhishen et al., 1999). The above soluble extract (1 mL) or a standard solution of catechin (0.5 mL) was mixed with 4 mL of water followed by addition of 0.3 mL of 5% (m/v) NaNO_2_, 0.3 mL of 10% (m/v) AlCl_3_ (after 5 min) and 2 mL of 1 M NaOH (1 min later). The volume was adjusted with water to 10 mL. The absorbance was measured spectrophotometrically at a wavelength of 510 nm. Total flavonoid content was expressed as catechin equivalent (CE) per leaf fresh weight.

### Statistical Analyses

All experiments were repeated at least three times. Statistical analyses were performed using Prism 5.0 (GraphPad Software). Two-way ANOVA analyses were followed by a Bonferroni post-test to compare plant lines within a treatment.

## Results

### Normoxia, Hypoxia, and Reoxygenation Experiment

Wild-type, AOX knockdown (RI9, RI29) and AOX overexpression (B7, B8) tobacco plants were compared in an experiment in which potted plants were subjected to either a 4 h normoxia (normal air) treatment, a 4 h hypoxia (0.001% oxygen, i.e., near anoxia) treatment, or a 4 h hypoxia treatment followed by a reoxygenation (normal air) treatment for 15 min or 120 min. Following treatment, leaf tissue was collected for a number of analyses, the results of which are described below.

**FIGURE 1:**
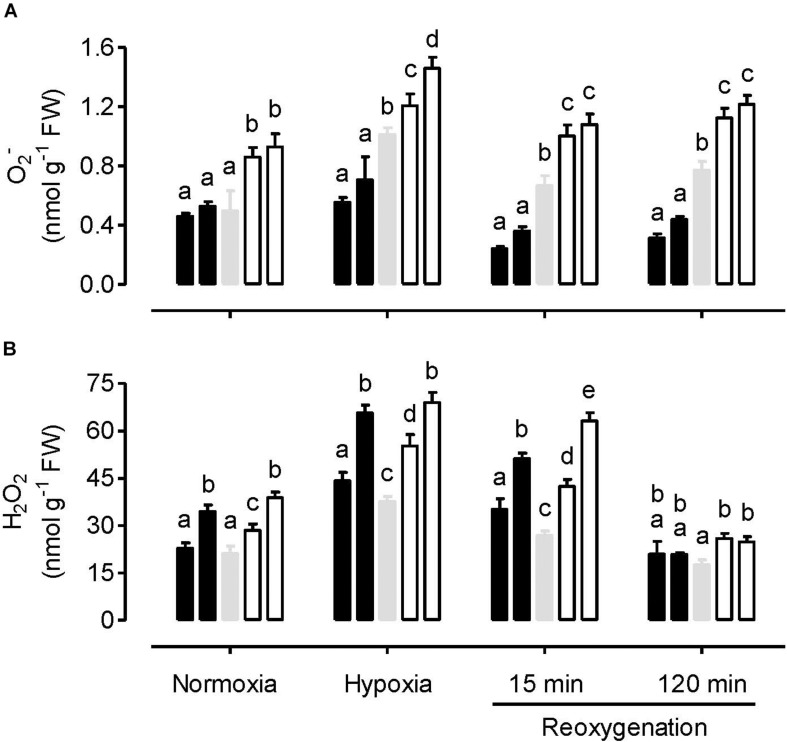
Leaf O_2_^–^ amount **(A)** and H_2_O_2_ amount **(B)** in tobacco plants treated under either normoxia conditions, hypoxia conditions (for 4 h), or reoxygenation conditions (for 15 or 120 min) following a 4 h hypoxia treatment. Data are presented for WT (gray bar), alternative oxidase overexpressors (B8, left closed bar; B7, right closed bar) and alternative oxidase knockdowns (RI9, left open bar; RI29, right open bar). Each experiment included single individuals for each plant line and treatment. Data are the mean ± SD of three independent experiments (*n* = 3). Within a treatment, plant lines not sharing a common letter are significantly different from one another (*P* < 0.05).

Under normoxia, leaf O_2_^–^ amount was similar between WT and overexpression plants, but significantly higher in the knockdowns (Figure 1A). In response to the hypoxia treatment, O_2_^–^ amount increased only slightly in the overexpressors, but more dramatically in the WT and knockdowns. Hence, following hypoxia, O_2_^–^ amount was significantly lower in the overexpressors and significantly higher in the knockdowns, compared to WT. These differences across plant lines were also evident during the reoxygenation period (Figure 1A).

The hypoxia treatment increased H_2_O_2_ amount in all plant lines, compared to the normoxia treatment (Figure 1B). Following reoxygenation, H_2_O_2_ amount declined in all plant lines, particularly by the 120 min time point, when amounts had returned to at or below that measured following the normoxia treatment. However, unlike the results with O_2_^–^, the relationship between H_2_O_2_ amount and AOX amount across the plant lines was complex, regardless of the treatment conditions. Under all conditions, the knockdowns had significantly higher H_2_O_2_ amount than WT. This was particularly evident following hypoxia and at the early stage (15 min) of reoxygenation. However, the AOX overexpressors also had higher H_2_O_2_ amounts than WT following hypoxia and after 15 min of reoxygenation (Figure 1B).

**FIGURE 2:**
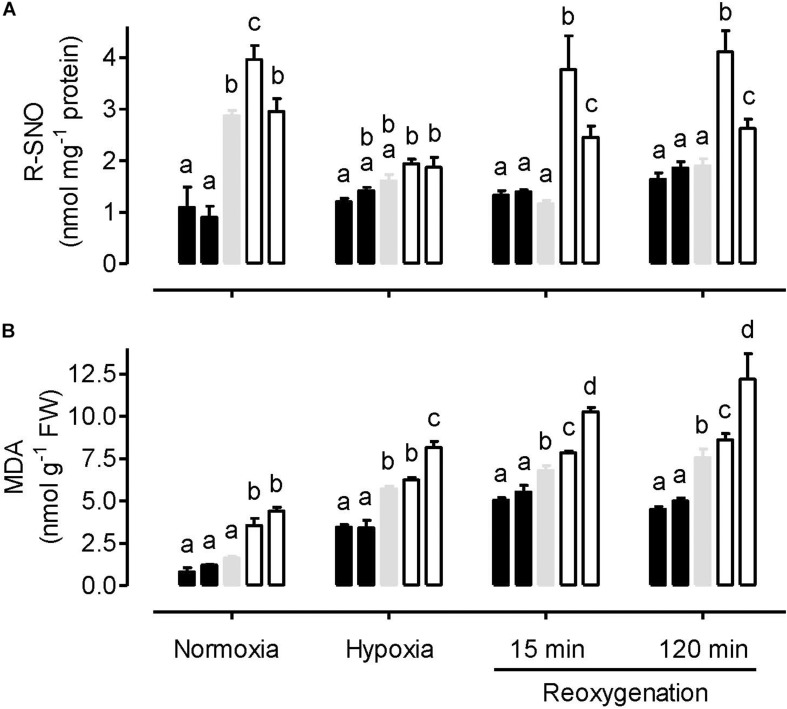
Leaf R-SNO amount **(A)** and MDA amount **(B)** in tobacco plants treated under either normoxia conditions, hypoxia conditions (for 4 h), or reoxygenation conditions (for 15 or 120 min) following a 4 h hypoxia treatment. Data are presented for WT (gray bar), alternative oxidase overexpressors (B8, left closed bar; B7, right closed bar) and alternative oxidase knockdowns (RI9, left open bar; RI29, right open bar). Each experiment included single individuals for each plant line and treatment. Data are the mean ± SD of three independent experiments (*n* = 3). Within a treatment, plant lines not sharing a common letter are significantly different from one another (*P* < 0.05).

Under normoxia, the amount of protein *S*-nitrosylation (R-SNO) was significantly lower in the AOX overexpressors, compared to the WT and knockdowns (Figure 2A). R-SNO amount was significantly higher in one knockdown line (RI9) compared to WT, but WT and RI29 showed similar R-SNO amount. In response to hypoxia, R-SNO amount decreased in the WT and knockdown plants, while increasing slightly in the overexpressors. Under these conditions, R-SNO amount was only slightly lower in the overexpressors and slightly higher in the knockdowns, compared to WT. Compared to the hypoxia treatment, there was little change in the R-SNO amount in WT and overexpression plants following reoxygenation. However, the R-SNO amount in knockdown plants increased during reoxygenation. Hence, at both the 15 and 120 min time points, R-SNO amounts were significantly higher in the knockdowns than other plant lines (Figure 2A).

The amount of lipid peroxidation (MDA equivalents) was lowest in the normoxia-treated plants. Under this condition, WT and overexpression plants displayed similar amounts, while the knockdowns displayed significantly higher amounts (Figure 2B). The hypoxia treatment increased MDA amount in all plant lines, compared to normoxia. Now, MDA amounts were significantly lower in the overexpressors, compared to WT. MDA amounts were higher in the knockdowns than WT, although this result was only significant in the case of RI29. Re-oxygenation increased MDA amounts further across all plant lines. At both time points following reoxygenation, MDA amounts were significantly lower in the overexpressors and significantly higher in the knockdowns, compared to WT (Figure 2B).

**FIGURE 3:**
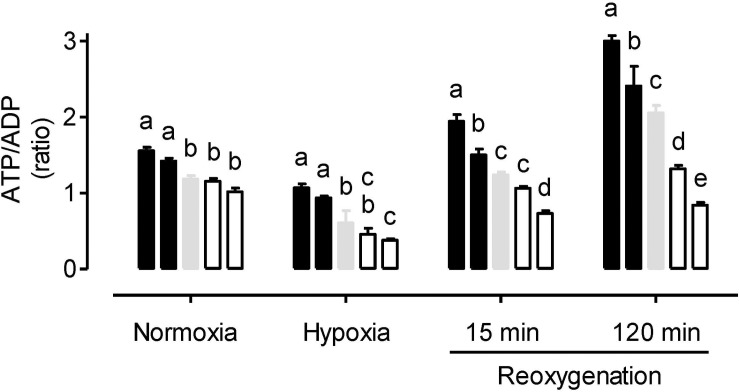
Leaf ATP/ADP ratio in tobacco plants treated under either normoxia conditions, hypoxia conditions (for 4 h), or reoxygenation conditions (for 15 or 120 min) following a 4 h hypoxia treatment. Data are presented for WT (gray bar), alternative oxidase overexpressors (B8, left closed bar; B7, right closed bar) and alternative oxidase knockdowns (RI9, left open bar; RI29, right open bar). Each experiment included single individuals for each plant line and treatment. Data are the mean ± SD of three independent experiments (*n* = 3). Within a treatment, plant lines not sharing a common letter are significantly different from one another (*P* < 0.05).

Under normoxia, the ATP/ADP ratio was similar between the WT and AOX knockdown plants, but slightly higher in the overexpressors (Figure 3). In response to hypoxia, the ATP/ADP ratio decreased in all plant lines. The ratio remained significantly higher in the overexpressors compared to WT. Further, the knockdowns now displayed lower ATP/ADP ratios than the WT, although this difference was only significant in the case of RI29. Re-oxygenation increased the ATP/ADP ratio of all plant lines, but also magnified the differences in ATP/ADP ratio between the plant lines. Particularly by the 120 min time point of reoxygenation, the ATP/ADP ratio was much higher in the overexpressors and much lower in the knockdowns, compared to WT (Figure 3). Interestingly, while the ATP/ADP ratio of the knockdowns recovered to values similar to that seen in the normoxia treatment, the WT, and overexpression plants now displayed ATP/ADP ratios that were much higher than measured during the normoxia treatment.

**FIGURE 4:**
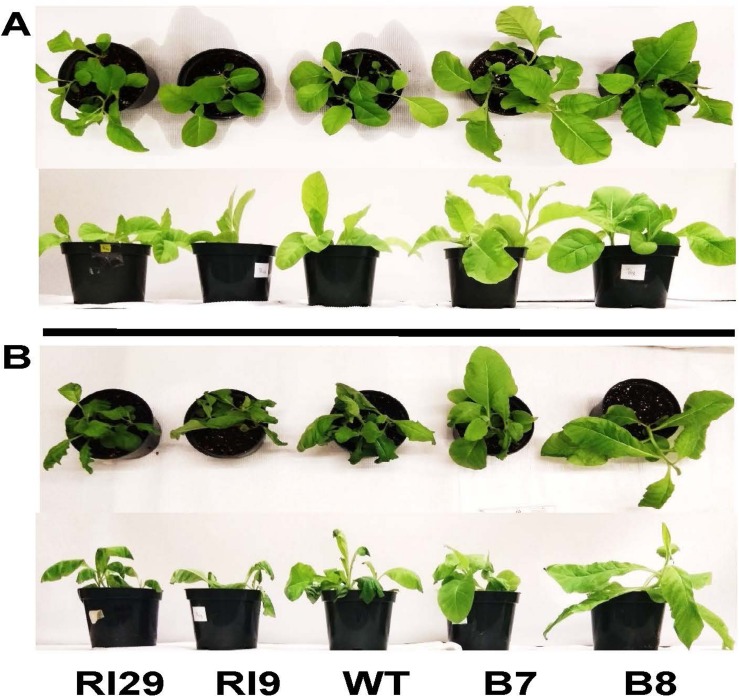
Typical appearance of wild-type (WT), AOX knockdown (RI9, RI29), and AOX overexpression (B7, B8) plants following either the normoxia **(A)** or 4 h hypoxia **(B)** treatment.

Interestingly, following the hypoxia treatment, the overexpression plants were more visibly upright, healthy and robust than the WT and knockdown plants, an observation that may relate to some of the above factors (such as energy status) but which requires further investigation (Figure 4).

### Nitric Oxide Emission From Hypoxia Treated Leaves

**FIGURE 5:**
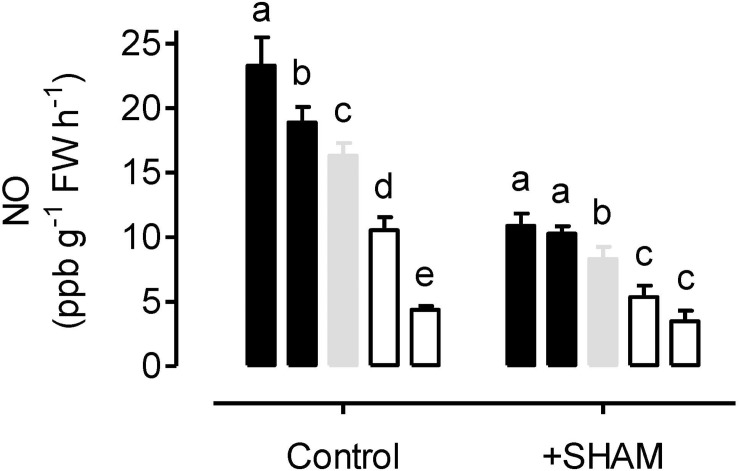
Nitric oxide (NO) emission rates from tobacco leaves in the presence of 50 mM sodium nitrate (control) or 50 mM sodium nitrate plus 5 mM SHAM. NO emission was measured in an atmosphere containing approximately 0.001% oxygen. Data are presented for WT (gray bar), alternative oxidase overexpressors (B8, left closed bar; B7, right closed bar) and alternative oxidase knockdowns (RI9, left open bar; RI29, right open bar). Each experiment included single individuals for each plant line and treatment. Data are the mean ± SD of three independent experiments (*n* = 3). Within a treatment, plant lines not sharing a common letter are significantly different from one another (*P* < 0.05).

Nitric oxide emission rates were measured from leaves incubated in nitrate solution and in an atmosphere containing 0.001% oxygen. Under these hypoxic conditions, NO emission rate was significantly higher in AOX overexpressors and significantly lower in AOX knockdowns, compared to WT (Figure 5). Further, tungstate treatment could dramatically lower NO production in all plant lines, while KCN treatment ceased NO production in all plant lines (data not shown).

When leaves were pre-treated with the AOX inhibitor SHAM, the rates of NO emission decreased in all plant lines (Figure 5). However, the absolute decrease in NO emission rate was greatest in the overexpressors (particularly B8), and least in the knockdowns (particularly RI29), with WT plants showing an intermediate response.

For three of the plant lines, NO emission was also measured at 0.1% O_2_. The absolute rates (in units of ppb NO g^–1^ FW h^–1^) were: B7, 5.69; WT, 5.65; and RI9, 3.87. Overall, these rates are much lower than measured at 0.001% O_2_ (compare to Figure 5). Also, while the WT and overexpressor display similar rates under these conditions, the rate in the knockdown remains significantly lower than in the other plant lines. In 3% oxygen or in ambient air, no NO emission could be detected by the chemiluminescence method.

### Aconitase Activity in Normoxia and Hypoxia Treated Plants

**FIGURE 6:**
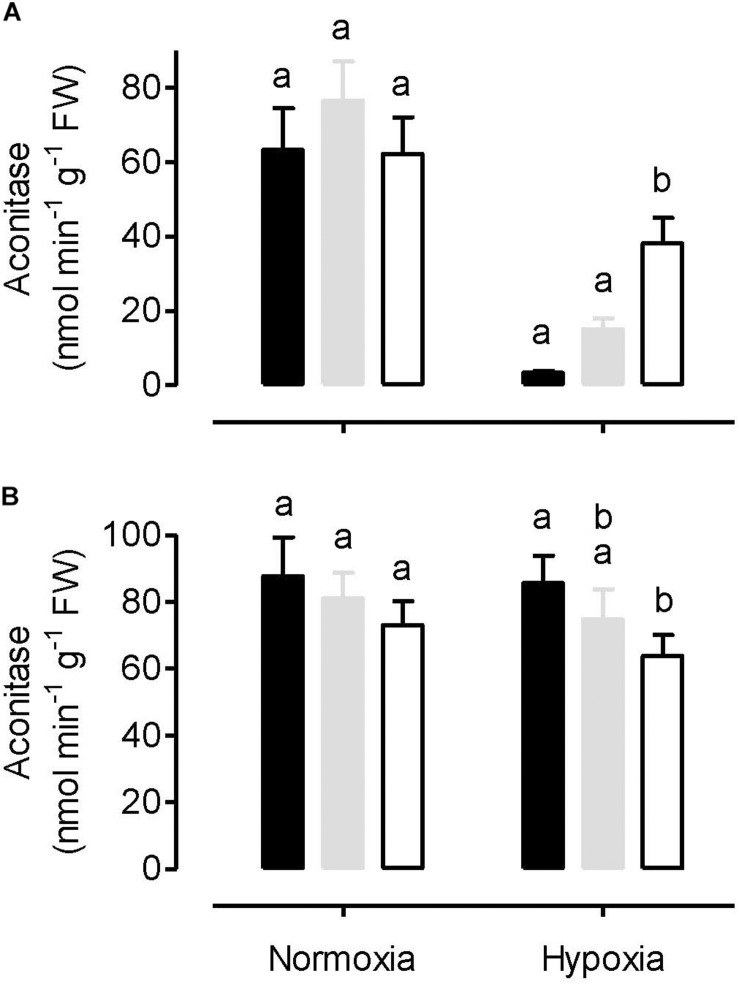
Leaf mitochondrial aconitase activity **(A)** and leaf cytosolic aconitase activity **(B)** in tobacco plants following either a normoxia or 3 h hypoxia treatment. Data are presented for WT (gray bar), alternative oxidase overexpressor (B7, closed bar), and alternative oxidase knockdown (RI9, open bar) plants. Each experiment included single individuals for each plant line and treatment. Data are the mean ± SD of three independent experiments (*n* = 3). Within a treatment, plant lines not sharing a common letter are significantly different from one another (*P* < 0.05).

Both leaf cytosolic aconitase and leaf mitochondrial aconitase activities were measured in plants following a normoxia or hypoxia (3 h, 0.001% O_2_) treatment. Under normoxia, the mitochondrial (Figure 6A) and cytosolic (Figure 6B) activities were comparable to one another, and each activity was similar between the WT, AOX knockdown (RI9) and AOX overexpression (B7) plants. The hypoxia treatment had a relatively small impact on cytosolic aconitase activity, decreasing by 13% in RI9, 8% in WT and just 2% in B7 (Figure 6B). However, the hypoxia treatment led to substantial losses of mitochondrial aconitase activity (Figure 6A). Further, this loss was most severe in the overexpressor (95% decrease in activity compared to normoxia) and least severe in the knockdown (39% decrease in activity), with the WT showing an intermediate response (80% decrease in activity).

### Metabolite Amounts in Normoxia and Hypoxia Treated Plants

**FIGURE 7:**
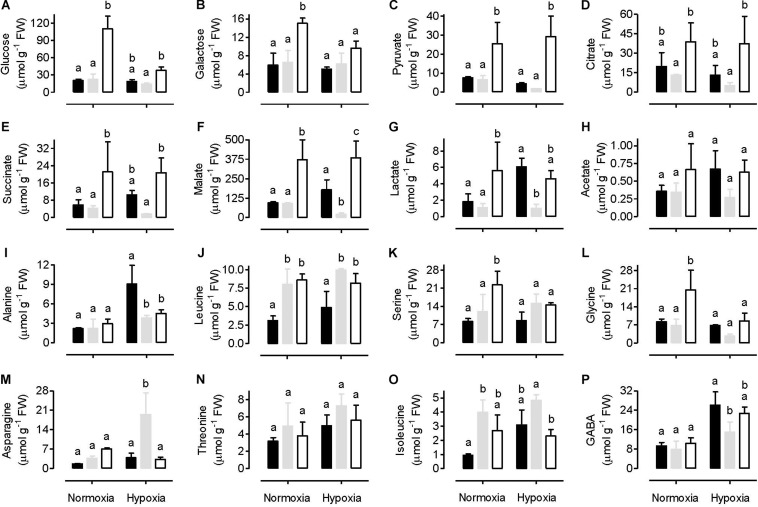
Leaf metabolite amounts in tobacco plants following either a normoxia or 3 h hypoxia treatment. Data are presented for WT (gray bar), alternative oxidase overexpressor (B7, closed bar) and alternative oxidase knockdown (RI9, open bar) plants. The metabolites include various sugars **(A,B)**, organic acids **(C–H)**, and amino acids **(I–P)**. Each experiment included single individuals for each plant line and treatment. Data are the mean ± SD of three independent experiments (*n* = 3). Within a treatment, plant lines not sharing a common letter are significantly different from one another (*P* < 0.05).

A range of primary metabolites (including two sugars, six organic acids, and eight amino acids) were identified and quantified using a ^1^H-NMR metabolomics technique. Under normoxia conditions, the amount of sugars (glucose and galactose) and organic acids (lactate, acetate, pyruvate, citrate, succinate and malate) in leaves were similar between the WT and AOX overexpressor (B7) (Figures 7A–H). In most cases, however, these metabolites were significantly higher in the AOX knockdown (RI9) than other plant lines. In WT plants, hypoxia had either little impact on the amount of these metabolites (in the case of glucose, galactose, lactate and acetate) or reduced their amounts by half or more (in the case of pyruvate, citrate, succinate and malate). In the knockdown, hypoxia reduced the amounts of glucose and galactose, while organic acid amounts were unaffected. Hence, the knockdown maintained higher organic acid amounts in leaves than WT under both the normoxia and hypoxia conditions (Figures 7C–H). The AOX overexpressor showed a different pattern following hypoxia. Sugar amounts were unchanged by hypoxia and were similar to WT amounts under hypoxia. Succinate, malate, lactate and acetate increased in the overexpressor under hypoxia and were now higher than in the WT under hypoxia. Finally, pyruvate and citrate showed a small decline in leaves following hypoxia, similar to that seen in the WT response to hypoxia (Figures 7A–H).

Under normoxia, the amount of most amino acids measured was similar between the WT and AOX knockdown, but serine and glycine amounts were significantly higher in the knockdown (Figures 7I–P). The amount of several amino acids was also similar between the WT and overexpressor under normoxia, but leucine and isoleucine were significantly lower in the overexpressor than WT (Figures 7I–P). Following hypoxia, serine and glycine amounts decreased in the knockdown and were now similar to the other plant lines (Figures 7K,L). While alanine tended to increase in all plant lines in response to hypoxia, the increase was particularly pronounced in the overexpressor, such that its alanine amount was now significantly higher than the other plant lines. In response to hypoxia, asparagine increased in the WT, but this response was not seen in either the overexpressor or knockdown plants (Figure 7M). The amount of GABA increased in all plant lines in response to hypoxia and was significantly higher in the overexpressor than WT (Figure 7P).

### Antioxidant Amounts in Normoxia and Hypoxia Treated Plants

**FIGURE 8:**
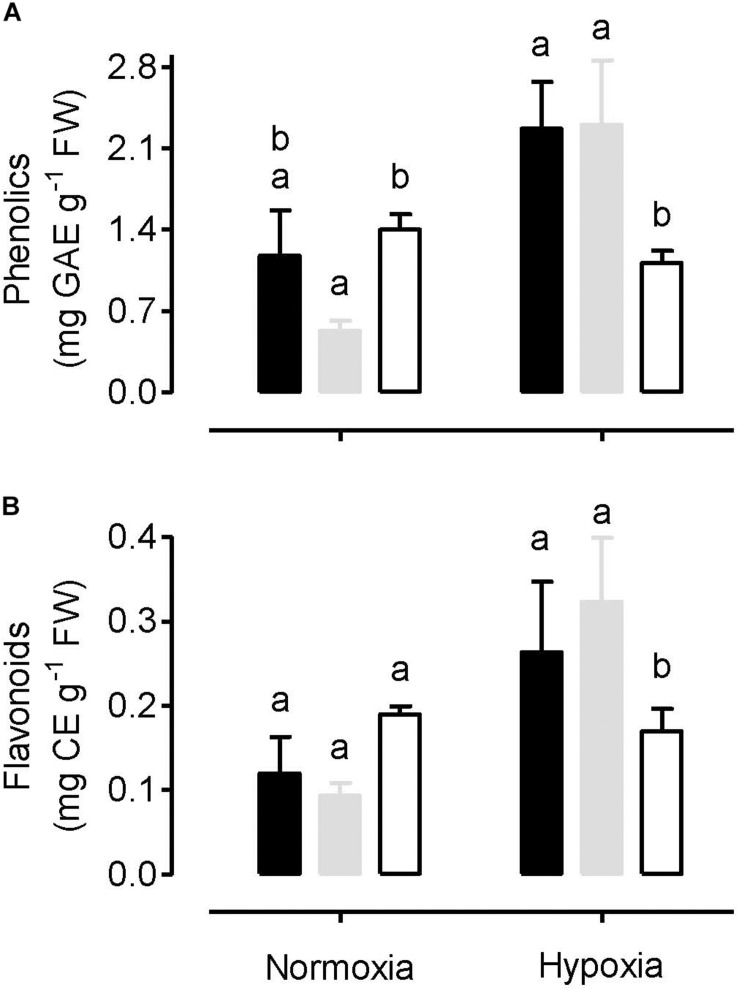
Total amount of leaf phenolics **(A)** and total amount of leaf flavonoids **(B)** in tobacco plants following either a normoxia or 3 h hypoxia treatment. Data are presented for WT (gray bar), alternative oxidase overexpressor (B7, closed bar) and alternative oxidase knockdown (RI9, open bar) plants. Each experiment included single individuals for each plant line and treatment. Data are the mean ± SD of three independent experiments (*n* = 3). Within a treatment, plant lines not sharing a common letter are significantly different from one another (*P* < 0.05).

The amount of total leaf phenolics and total leaf flavonoids were measured in plants following a normoxia or hypoxia (3 h, 0.001% O_2_) treatment. Under normoxia, phenolics (Figure 8A) and flavonoids (Figure 8B) were slightly lower in WT plants, compared to either the AOX knockdown or overexpression plants. However, these differences were only significant in the case of the phenolics amount in the knockdown. Following the hypoxia treatment, the amount of phenolics and flavonoids increased in both the WT and overexpression plants, but not in the knockdown. Hence, following hypoxia, the content of phenolics and flavonoids was significantly greater in the WT and overexpressor, compared to the knockdown (Figure 8).

## Discussion

We investigated the significance of mitochondrial AOX under different oxygen conditions by comparing WT tobacco plants to AOX knockdown and AOX overexpression plants following either: (1) a normoxia (normal air) treatment; (2) a short-term (3–4 h) hypoxia (near-anoxia) treatment; (3) a reoxygenation period (of 15 or 120 min) following a hypoxia treatment. The discussion below deals with each of these treatments in sequence.

### Normoxia

Under normoxia conditions, knockdown of AOX increased the leaf amount of O_2_^–^ (Figure 1A). This is evidence that AOX respiration is important in controlling ROS generation by the mitochondrial ETC. The results confirm previous fluorescent imaging studies in tobacco leaf using the MitoSOX mitochondrial O_2_^–^ indicator (Cvetkovska and Vanlerberghe, 2012a). In that study, knockdown of AOX increased the amount of mitochondria-localized O_2_^–^ relative to WT, while overexpression of AOX had little effect, similar to the results seen here. Interestingly, the pattern of O_2_^–^ amount across plant lines was mirrored by the pattern of MDA amount across plant lines, which was also elevated in the knockdowns, but unaffected in the overexpressors, relative to WT (Figure 2B). These results suggest that increased ROS generation by the mitochondrial ETC can increase the amount of oxidative damage (lipid peroxidation) in leaf tissues. Overall, these results are consistent with a large body of evidence indicating that AOX respiration reduces mitochondrial ETC-generated ROS under normal oxygen conditions (Purvis, 1997; Maxwell et al., 1999; Parsons et al., 1999; Giraud et al., 2008; Smith et al., 2009; Cvetkovska and Vanlerberghe, 2012a).

Similar to O_2_^–^, knockdown of AOX also increased the leaf amount of H_2_O_2_ under normoxia (Figure 1B). However, the relationship between AOX amount and H_2_O_2_ amount appears more complex since overexpression of AOX also increased leaf H_2_O_2_ amount, at least in one of the two overexpressors (B7). One possibility is that the aberrant amount of ROS generation by the mitochondrial ETC can signal changes in the capacity of ROS-scavenging systems within the cell, leading to unexpected changes in H_2_O_2_ amount. This was reported before in tobacco plants with altered AOX amount. In one study, overexpression of AOX dampened the induction of scavenging systems, resulting paradoxically in an increase in H_2_O_2_ amount (Pasqualini et al., 2007) while in another study, knockdown of AOX was shown to magnify the induction of scavenging systems (Wang et al., 2011). Overall, such results suggest that, in evaluating the influence of AOX respiration on ROS generation by the mitochondrial ETC, the likely most unambiguous approach is to measure the primary product of that process (O_2_^–^) rather than H_2_O_2_, the amount of which will depend upon the secondary processes associated with its formation and scavenging. Interestingly, the overall pattern of H_2_O_2_ amount across plant lines mirrored the pattern of antioxidants (phenolics, flavonoids), which also tended to be higher in both RI9 and B7, relative to WT (Figure 8). These results suggest that the production of these secondary metabolites in tobacco leaf is responsive to the H_2_O_2_-load on the cell.

Previously, it was shown that AOX knockdown tobacco plants under normoxia have higher amounts of leaf mitochondria-localized NO, compared to WT plants (Cvetkovska and Vanlerberghe, 2012a). Alber et al. (2017) provided evidence that the increase in NO was due to an increased leak of mitochondrial ETC electrons to nitrite, possibly at Complex III, as postulated earlier (Igamberdiev et al., 2010). One important consequence of NO is that it can result in the S-nitrosylation of target proteins (Baudouin, 2011; Zaffagnini et al., 2016). Indeed, the amount of protein *S*-nitrosylation (R-SNO) did differ across the plant lines under normoxia (Figure 2A). Amounts were lowest in the overexpressors and highest in the knockdowns, consistent with the idea that AOX respiration can act to reduce the mitochondrial ETC generation of NO under normoxia.

Electron flow from ubiquinol to oxygen through AOX is not coupled to proton translocation from matrix to inter-membrane space. Hence, knockdown of AOX could enhance overall proton translocation and oxidative phosphorylation by diverting additional electrons toward Complex’s III and IV. Similarly, overexpression of AOX might decrease overall proton translocation and oxidative phosphorylation by diverting electrons away from Complex’s III and IV and toward AOX. To examine these possibilities, we measured the energy status (ATP/ADP ratio) of the different plant lines. Paradoxically, the ATP/ADP ratio was significantly higher in the AOX overexpressors than WT (Figure 5). It was also slightly lower in the knockdowns than WT, although this result was not significant. These results suggest that increased AOX respiration can improve leaf energy status despite its non-energy conserving nature. More investigation is required to uncover the mechanistic basis of this observation. One possibility is that aberrant amounts of NO in the transgenic lines, as discussed above, is partly responsible. NO is a potent inhibitor of cyt oxidase, so higher amounts of NO in the mitochondrion (knockdown lines) could be acting to restrict cyt oxidase activity (hence lowering the ATP/ADP ratio), while lower amounts of NO in the mitochondrion (overexpression lines) could result in increased cyt oxidase activity (hence increasing the ATP/ADP ratio). Interestingly, it was previously shown that lower NO in barley roots following overexpression of a non-symbiotic phytoglobin did increase respiration rates under normoxia (Gupta et al., 2014).

Of the two sugars and six organic acids analyzed, all but one of these were significantly higher in amount in AOX knockdown than WT plants under normoxia (Figures 7A–H). The exception was acetate, which, while still approximately twofold higher in the knockdown than the WT, was not significantly different in amount. Two (of eight) amino acids analyzed (serine and glycine) were also significantly higher in the knockdown than WT, while the others were similar to WT (Figures 7I–P). One interpretation of these results is that lack of AOX respiration can significantly impede upstream respiratory carbon metabolism, resulting in the accumulation of carbon intermediates. Likely, the lack of AOX primarily restricts ETC activity (as suggested by the higher amounts of O_2_^–^, Figure 1A) and this restriction then secondarily backs up carbon metabolism, particularly organic acid metabolism by the tricarboxylic acid (TCA) cycle. Of particular interest is the accumulation of pyruvate and lactate (Figures 7C,G). Pyruvate is an important biochemical activator of AOX (Selinski et al., 2018). Hence, pyruvate accumulation is thought to activate AOX in order to stimulate TCA cycle carbon flow, and hence draw down the pyruvate amount (Vanlerberghe et al., 1995). Consistent with this model, our results show that, in the absence of AOX, pyruvate does accumulate. A consequence of this accumulation is the overflow of pyruvate into fermentation pathways, such as seen here by the accumulation of lactate. On the other hand, the large majority of metabolites tested did not differ significantly between the WT and overexpressor under normoxia, except for two amino acids (leucine and isoleucine) that were less abundant in the overexpressor. These plants also displayed similar O_2_^–^ amount to the WT (Figure 1A).

In summary, AOX does significantly influence respiratory metabolism in tobacco leaf under normoxia. A lack of AOX disrupted mitochondrial ETC activity, increasing O_2_^–^ generation, and restricting respiratory carbon flow. Overexpression of AOX had less pervasive effects, likely due in part to the fact that the protein is subject to tight post-translational biochemical controls over its activity (Guy and Vanlerberghe, 2005). However, overexpression did lower R-SNO amounts relative to WT, suggestive of less NO generation by the mitochondrial ETC, and it did increase the ATP/ADP ratio relative to WT, suggestive of higher cyt oxidase activity. These two observations may be linked (Gupta et al., 2014).

### Hypoxia

Alternative oxidase has a much lower affinity for oxygen than does cyt oxidase. The *K*_*m*_ for oxygen is estimated at 10 μM for AOX, compared to 0.08–0.16 μM for cyt oxidase (Hoshi et al., 1993; Millar et al., 1994; Affourtit et al., 2001). While this difference might be expected to limit AOX activity at low oxygen concentrations, others factors may actually favor AOX activity under these conditions. First, AOX is subject to biochemical controls (Millar et al., 1993; Umbach and Siedow, 1993; Vanlerberghe et al., 1995; Selinski et al., 2018) that may favor AOX activity under hypoxia. The more reducing conditions [higher NAD(P)H/NAD(P) ratios] typical of hypoxia will favor reduction of an AOX regulatory disulfide bond to its component sulfhydryls. These sulfhydryls can then interact with 2-oxoacids, most notably pyruvate, resulting in a strong activation of AOX activity. Under hypoxia, a stimulation of glycolysis combined with a slowing of the TCA cycle might increase pyruvate amount, as reported in some studies (Rocha et al., 2010; António et al., 2016). Second, under hypoxia, cyt oxidase displays a nitrite reductase activity that generates NO from nitrite (Poyton et al., 2009). This NO can then inhibit the oxidase activity of the enzyme (Poyton et al., 2009). On the other hand, AOX is NO-resistant (Millar and Day, 1997). The inhibition of cyt oxidase by NO slows oxygen consumption such that, even in an atmosphere of almost pure nitrogen (0.001% oxygen), the oxygen concentration in the cell can remain in the micromolar range (Benamar et al., 2008; Gupta et al., 2009, 2018). Hence, under hypoxia, there could be a shift in the consumption of oxygen away from cyt oxidase and toward AOX, despite the lower affinity for oxygen of AOX compared to cyt oxidase. Another potentially confounding factor is the possibility of nitrite reduction to NO by AOX itself under hypoxia. Initially, this hypothesis was based on pharmacological evidence, where the AOX inhibitor SHAM was shown to decrease NO emission rates from tobacco leaf under hypoxia (Planchet et al., 2005). More recently, additional support for this hypothesis has come from a study showing that *Arabidopsis* plants overexpressing AOX display increased NO emission rates under hypoxia (Vishwakarma et al., 2018).

As might be expected, the severe hypoxia treatment reduced the ATP/ADP ratio of all the plant lines, relative to normoxia (Figure 3). However, the relatively small differences in energy status between the plant lines seen under normoxia were magnified by the hypoxia treatment. These results establish an important role for AOX activity in the maintenance of energy status under hypoxia. One possibility is that AOX becomes a key electron sink to maintain electron flow to any available oxygen, under conditions when NO is inhibiting cyt oxidase (see more below). Even with AOX activity alone (i.e., complete inhibition of cyt oxidase), proton translocation could still proceed at Complex I and allow for some oxidative phosphorylation. Another indication that AOX is acting as a key electron sink under these conditions are the results regarding O_2_^–^ amount. Following hypoxia, O_2_^–^ amounts are much lower in the overexpressors and much higher in the knockdowns, relative to WT, an indication that AOX is providing a means to prevent over-reduction of the ETC under these conditions (Figure 1A). Consistent with this, while oxidative damage (MDA amount) increased in all plant lines following hypoxia, this damage was least abundant in the overexpressors and most abundant in the knockdowns, with WT showing an intermediate response (Figure 2B).

Under hypoxia, NO emission rates were highest in the overexpressors, and lowest in the knockdowns, with WT showing an intermediate response (Figure 5). The large difference across plant lines may provide some explanation why R-SNO amounts following hypoxia increased slightly in the overexpressors (relative to normoxia) but decreased strongly in the WT and knockdowns (Figure 2A). The positive relationship between NO emission rates and AOX amount across plant lines is consistent with the hypothesis that AOX is able to produce NO from nitrite under hypoxia (Planchet et al., 2005; Vishwakarma et al., 2018). If this is the case, AOX could be contributing to the maintenance of energy status (ATP/ADP ratio) not so much by maintaining electron flow to oxygen (as discussed above), but rather by contributing to the phytoglobin-NO cycle. In this cycle, nitrite (rather than oxygen) acts as a terminal electron acceptor in the mitochondrial ETC, allowing for the maintenance of some proton translocation and oxidative phosphorylation. The NO then leaks from the mitochondrion and the nitrogen is cycled back to nitrate using non-symbiotic class 1 phytoglobins with extremely high affinity for oxygen (Igamberdiev and Hill, 2004; Stoimenova et al., 2007; Gupta et al., 2011). The cycle depends upon cyt oxidase (to act as a nitrite reductase) and nitrate reductase (to convert nitrate back to nitrite). In the current study, either the cyt oxidase inhibitor KCN or the nitrate reductase inhibitor tungstate dramatically reduced NO emission rates under hypoxia, indicating that this cycle is active. However, the AOX inhibitor SHAM could also reduce NO emission rates significantly and this inhibition was most pronounced in the overexpressors and lease pronounced in the knockdowns (Figure 5). This is again consistent with the hypothesis that AOX can produce NO. However, results with SHAM should be considered with caution since its specificity is quite low, allowing it to inhibit peroxidases and other redox proteins as well (Rich et al., 1978).

While aberrant amounts of NO emission in the knockdown and overexpression plants (Figure 5) is consistent with AOX being capable of nitrite reduction to NO under hypoxia, an alternative explanation should also be considered. NO can be scavenged by its very fast reaction with O_2_^–^ to produce peroxynitrite, which in turn can be detoxified by mitochondrial peroxidases (de Oliveira et al., 2008; Wulff et al., 2009; Quijano et al., 2016). Since AOX influences the generation of mitochondrial O_2_^–^, it could affect the rate of mitochondrial NO scavenging by this reaction, thereby influencing the rate of leaf NO emission. If AOX knockdowns produce more O_2_^–^ than WT plants under hypoxia (Figure 1A), then their lower NO emission rates (Figure 5) could be due to more effective NO scavenging in the mitochondrion. Similarly, lower O_2_^–^ production in overexpressors would decrease NO scavenging, hence increasing NO emissions. Further, the lower NO emission rates in SHAM-treated plants (particularly the WT and overexpression plants) could be explained in this manner. Inhibition of AOX in these plants by SHAM would increase O_2_^–^ generation and hence NO scavenging.

As discussed earlier, under normoxia, knockdown of AOX altered the abundance of the majority of the metabolites measured in this study (9 out of 16 metabolites differed significantly from WT), while overexpression of AOX had relatively little effect (2 out of 16 metabolites differed significantly from WT) (Figure 7). However, following hypoxia, the number of metabolites with significantly altered abundance relative to WT was similar between the knockdown (7 out of 16 metabolites) and overexpression (6 out of 16 metabolites) plants. These results suggest that AOX overexpression was more disruptive to carbon metabolism under hypoxia than under normoxia. For example, while lactate and alanine were similar between the WT and overexpression plants under normoxia, they were higher in the overexpressor than WT following hypoxia (Figures 7G,I).

One site of disruption of carbon metabolism in the overexpressor following hypoxia was at mitochondrial aconitase. The overexpressor retained only 5% of this activity following hypoxia (relative to normoxia), while the WT and knockdown retained 20 and 61%, respectively (Figure 6A). Aconitase is susceptible to oxidative stress (Sweetlove et al., 2002; Armstrong et al., 2004; Quijano et al., 2016), but if this were responsible for the losses of activity seen here, then one would predict the greatest losses to have occurred in the knockdown, which in fact saw the least loss of activity. However, aconitase may also be susceptible to inactivation by NO (Navarre et al., 2000; Gupta et al., 2012). Indeed, the exaggerated loss of activity in the overexpressor relative to WT corresponds with its higher rate of NO emission under hypoxia (Figure 5). Similarly, the greater retention of activity in the knockdown relative to WT corresponds with its lower rate of NO emission. These results provide compelling (yet circumstantial) evidence that, under hypoxia, mitochondrial aconitase may be susceptible to inactivation by NO. On the other hand, cytosolic aconitase activity decreased only slightly in response to hypoxia, and this decline was similar across plant lines (Figure 6B).

In summary, AOX clearly remains relevant at very low oxygen concentrations, despite its much lower affinity for oxygen than cyt oxidase. AOX activity under these conditions supports energy generation, at a time when mitochondria-generated NO is inhibiting cyt oxidase. AOX prevents excessive nitro-oxidative stress under these conditions.

### Reoxygenation

Within 15 min of reoxygenation, leaf energy status (i.e., ATP/ADP ratio) of all plant lines improved relative to hypoxia, suggestive of a rapid recovery of ETC activity (Figure 3). Nonetheless, the differences in ATP/ADP ratio across plant lines persisted, suggesting that AOX was contributing positively to this recovery of energy status during reoxygenation. In fact, by 120 min, those plants containing AOX (WT and overexpression plants) had ATP/ADP ratios that now far exceeded those measured in the normoxia treatment, while this was not the case in the knockdowns. Nonetheless, O_2_^–^ amounts remained low in the WT and overexpressors (particularly the overexpressors), while the knockdown plants showed high amounts of O_2_^–^. In fact, the differences in O_2_^–^ amount across plant lines during the reoxygenation period were as great as, or greater, than seen under hypoxia. As a result, the differences in oxidative damage (MDA amount) across plant lines was greater following 120 min of reoxygenation than with any other treatment (Figure 2B). Overall, these results confirm that reoxygenation can elicit oxidative stress (Blokhina et al., 2003; Chang et al., 2012; Shingaki-Wells et al., 2014). Further, AOX provides a means to dampen this stress and improve the recovery of energy status.

Reoxygenation resulted in a spike in protein *S*-nitrosylation that was specific to the AOX knockdowns (Figure 2A). These results are consistent with previous studies indicating that, under normoxia, AOX respiration reduces NO generation by the ETC, by preventing over-reduction of Complex III (Cvetkovska and Vanlerberghe, 2012a, b; Alber et al., 2017).

In summary, reoxygenation represents a condition in which AOX appears particularly important in preventing excessive nitro-oxidative stress. In doing so, AOX improved the ability of leaf tissue to rapidly recover its energy status.

### Conclusion

The results show that AOX amount influences leaf carbon and energy metabolism under normoxia, hypoxia, and during a reoxygenation period following hypoxia. Under all these conditions, an optimal AOX amount was necessary to support metabolism and to prevent excessive nitro-oxidative stress.

## Data Availability Statement

The datasets generated for this study are available on request to the corresponding author.

## Author Contributions

JJ performed the experiments on normoxia, hypoxia, and reoxygenation including NO measurements, participated in discussion of results, and contributed to writing the manuscript. DC determined the aconitase activity, prepared the samples for metabolomics analysis, analyzed the results, and contributed to writing the manuscript. PV determined the phenolic and flavonoid content, and prepared the samples for metabolomics analysis. NB planned and supervised the experiments, analyzed the results, and contributed to writing the manuscript. GV developed the transgenic lines, analyzed the results, and contributed to writing the manuscript and shaping its final version. AI supervised the design, execution, and interpretation of the experiments, and prepared the manuscript for submission.

## Conflict of Interest

The authors declare that the research was conducted in the absence of any commercial or financial relationships that could be construed as a potential conflict of interest.
